# Collaborative Research Activities of the Arase and Van Allen Probes

**DOI:** 10.1007/s11214-022-00885-4

**Published:** 2022-06-21

**Authors:** Y. Miyoshi, I. Shinohara, S. Ukhorskiy, S. G. Claudepierre, T. Mitani, T. Takashima, T. Hori, O. Santolik, I. Kolmasova, S. Matsuda, Y. Kasahara, M. Teramoto, Y. Katoh, M. Hikishima, H. Kojima, S. Kurita, S. Imajo, N. Higashio, S. Kasahara, S. Yokota, K. Asamura, Y. Kazama, S.-Y. Wang, C.-W. Jun, Y. Kasaba, A. Kumamoto, F. Tsuchiya, M. Shoji, S. Nakamura, M. Kitahara, A. Matsuoka, K. Shiokawa, K. Seki, M. Nosé, K. Takahashi, C. Martinez-Calderon, G. Hospodarsky, C. Colpitts, Craig Kletzing, J. Wygant, H. Spence, D. N. Baker, G. D. Reeves, J. B. Blake, L. Lanzerotti

**Affiliations:** 1 Institute for Space-Earth Environmental Research, Nagoya University, Nagoya, 464-8601 Japan; 2 Institute of Space and Astronautical Science, Japan Aerospace Exploration Agency, Sagamihara, 252-5210 Japan; 3 Applied Physics Laboratory, The Johns Hopkins University, 11101 Johns Hopkins Rd, Laurel, MD 20723 USA; 4 Department of Atmospheric and Oceanic Sciences, University of California, Los Angeles, 7115 Math Sciences Bldg., Los Angeles, CA 90095 USA; 5 Faculty of Mathematics an Physics, Charles University, V Holesovickach 2, 18000 Prague, Czechia; 6 Dept. of Space Physics, Institute of Atmospheric Physics, Czech Academy of Sciences, Bocni II 1401, 14100 Prague, Czechia; 7 Graduate School of Natural Science and Technology, Kanazawa University, Kanazawa, 920-1192 Japan; 8 Graduate School of Engineering, Kyushu Institute of Technology, Kitakyusyu, 804-8550 Japan; 9 Graduate School of Science, Tohoku University, Sendai, 980-8578 Japan; 10 Research Institute for Sustainable Humanosphere, Kyoto University, Uji, 611-0011 Japan; 11 Graduate School of Science, Kyoto University, Kyoto, 606-8502 Japan; 12 Strategic Planning and Management Department, Japan Aerospace Exploration Agency, Tokyo, 101-8008 Japan; 13 Graduate School of Science, University of Tokyo, Tokyo, 113-0033 Japan; 14 Graduate School of Science, Osaka University, Toyonaka, 560-0043 Japan; 15 Institute of Astronomy and Astrophysics, Academia Sinica, No. 1, Sec. 4, Roosevelt Rd, Taipei, 10617 Taiwan; 16 Institute for Advanced Research, Nagoya University, Nagoya, 464-8601 Japan; 17 Department of Physics and Astronomy, University of Iowa, Van Allen Hall (VAN), Iowa City, IA 52242 USA; 18 Institute for the Study of Earth, Oceans, and Space, University of New Hampshire, 8 College Road, Durham, NH 03824 USA; 19 Laboratory for Atmospheric and Space Physics, University of Colorado, 3665 Discovery Drive, 600 UCB, Boulder, CO 80303 USA; 20 Inteligence & Space Reserarch Division, Los Alamos National Laboratory, PO Box 1663, Los Alamos, NM USA; 21 The Aerospace Corporation, P.O. Box 92957, Los Angeles, CA 90009-2957 USA; 22 Department of Physics, New Jersey Institute of Technology, Newark, NJ 07102 USA; 23 School of Physics and Astronomy, University of Minnesota, 116 Church St. SE, Minneapolis, MN 55455 USA

**Keywords:** Arase, Van Allen Probes, Inner magnetosphere, Radiation belts

## Abstract

This paper presents the highlights of joint observations of the inner magnetosphere by the Arase spacecraft, the Van Allen Probes spacecraft, and ground-based experiments integrated into spacecraft programs. The concurrent operation of the two missions in 2017–2019 facilitated the separation of the spatial and temporal structures of dynamic phenomena occurring in the inner magnetosphere. Because the orbital inclination angle of Arase is larger than that of Van Allen Probes, Arase collected observations at higher $L$-shells up to $L \sim 10$. After March 2017, similar variations in plasma and waves were detected by Van Allen Probes and Arase. We describe plasma wave observations at longitudinally separated locations in space and geomagnetically-conjugate locations in space and on the ground. The results of instrument intercalibrations between the two missions are also presented. Arase continued its normal operation after the scientific operation of Van Allen Probes completed in October 2019. The combined Van Allen Probes (2012-2019) and Arase (2017-present) observations will cover a full solar cycle. This will be the first comprehensive long-term observation of the inner magnetosphere and radiation belts.

## Introduction

The inner magnetosphere is a natural cavity wherein various types of charged particles are trapped by an intrinsic magnetic field. Different plasma and particle populations ranging in energy from a few electron volts (eV) to tens of megaelectron volts (MeV) coexist in the same region. These particles undergo different physical processes and are never stable, even at geomagnetically quiet times, owing to variations in the solar wind and instabilities within the inner magnetosphere (e.g., Ebihara and Miyoshi 2011; Li and Hudson 2019; Kanekal and Miyoshi 2021).

The inner magnetosphere is composed of several plasma and particle populations, ranging from the Earth’s ionosphere out to the plasmasphere and then farther out into the plasma sheet, radiation belt and ring current regions. The plasmasphere consists of cold and dense plasma, as does the ionosphere as the source of plasmaspheric plasma. The typical energy of the plasmaspheric plasma is lower than 1 eV. The typical ring current and plasma sheet energies range from a few kiloelectron volts (keV) to ∼100 keV; these more tenuous populations contribute to the ambient plasma pressure. The typical energies of radiation belt particles range from a few hundred keV to higher than 10 MeV (electrons) and 100 MeV (ions), which is the highest energy population in the geospace. Thus, plasma populations with a wide range of densities and energies, from a few eV to more than 10 MeV, coexist in the inner magnetosphere.

These energy regimes are directly or indirectly coupled with each other. Mass and energy are transported to other regions such as the outer magnetosphere and ionosphere. Thus, research efforts directed toward cross-energy and cross-regional couplings are required for a comprehensive understanding of the inner magnetosphere.

Many satellites have explored the inner magnetosphere. In the 1990s, the Combined Release and Radiation Effects Satellite (CRRES) observed the inner magnetosphere near the equatorial plane with an inclination angle of 18° and an apogee of 6.3 Re, CRRES thus covered $L$ = ∼3-7. The CRRES spacecraft carried a variety of instruments measuring plasma waves and particles, which greatly enhanced our understanding of the dynamics of the inner magnetosphere. Other satellites that obtained similar observations of particles and field variations in the inner magnetosphere include the Equator-S satellite and more recently, the five Time History of Events and Macroscale Interactions satellites, three of which have an apogee of ∼12 Re (Angelopoulos 2008).

Comprehensive observations of plasma/particles and fields/waves in the inner magnetosphere were achieved for the first time with the launch of NASA’s Van Allen Probes in 2012 (Mauk et al. 2013). The twin Van Allen Probes satellites with an apogee of ∼6 Re have comprehensive instruments to measure plasma and particles in a wide energy range (1 eV to > 100 MeV) and fields as well as waves over a wide frequency range (DC to 400 kHz) for a period of 7 years (e.g., Baker et al. 2019; Li and Hudson 2019). The results are summarized in the other chapters.

Multi-point observations are essential for a comprehensive understanding of the inner magnetosphere. Several types of plasma waves play a key role in the transport, acceleration, and loss of radiation belt electrons [see e.g., review by Ripoll et al. 2020]. Whistler-mode chorus waves contribute to both acceleration and loss of relativistic electrons on the dawn side outside the plasmapause, whereas whistler-mode hiss waves cause pitch-angle scattering within the plasmasphere. Electromagnetic ion cyclotron (EMIC) waves cause a strong scattering of MeV electrons, which are mainly observed on the dusk and noon sides. These wave–particle interactions often occur simultaneously at different local times and radial distances.

The causes and consequences of these processes are often observed in different locations. For example, pitch-angle scattering with whistler-mode waves occurs in the magnetosphere, and the resultant precipitation is observed at ionospheric altitudes. Considering this finding, it is possible to identify causal relationships using conjugate observations between satellite and low altitude CubeSat, balloon, and ground-based instruments. Thus, multi-point observations by multiple satellites and conjugate observations with ground-based stations are necessary to understand the dynamics of the inner magnetosphere.

The exploration of energization and radiation in geospace (ERG) /Arase satellite was facilitated by the Institute of Space and Astronautical Science (ISAS), Japan Aerospace Exploration Agency (JAXA). Nine scientific instruments onboard the satellite have been developed by ISAS/JAXA and at several universities and institutes in Japan and Taiwan. The satellite was successfully launched on December 20, 2016. After the commissioning phase operation, the satellite began its nominal operation at the end of March 2017 (Miyoshi et al. 2018a).

**Fig. 1:**
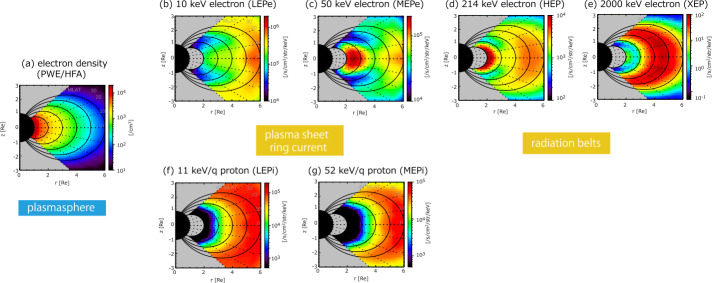
Average distributions of different plasma/protons in the inner magnetosphere. (a) electron density (PWE/HFA), (b) 10 keV electron flux (LEPe), (c) 50 keV electron flux (MEPe), (d) 214 keV electron flux (HEP), (e) 2000 keV electron flux (XEP), (f) 11 keV/q proton flux (LEPi), and (g) 52 keV/q proton flux (MEPi)

Figure 1 shows an overview of the plasma/electron populations of the inner magnetosphere obtained from Arase observations (April 2017–March 2020). Figure 1a shows the thermal plasma density based on plasma wave observations from the plasma wave experiment (PWE)/High Frequency Analyzer (HFA) instrument onboard the Arase satellite (Kasahara et al. 2018a; Kumamoto et al. 2018). The dense population at $L$ < ∼4 corresponds to the plasmasphere. Figure 1b and c show the ring current and plasma sheet electrons observed by Low-Energy Particle Experiments-Electron Analyzer (LEPe) (Kazama et al. 2017) and Medium-Energy Particle Experiments-Electron Analyzer (MEPe) instruments (Kasahara et al. 2018b). Figure 1d and e show sub-relativistic and relativistic electrons, located in the radiation belts observed by High-Energy Electron Experiments (HEP) (Mitani et al. 2018) and Extremely High-Energy Electron Experiment (XEP) instruments (Higashio et al. 2018). Figure 1f and g show the ring current and plasma sheet populations observed by Low-Energy Particle Experiments (LEPi) (Asamura et al. 2018) and Medium-Energy Particle Experiments—Ion Mass Analyzer (MEPi) (Yokota et al. 2017). For Fig. 1, the following time intervals (2017/05/26-2017/06/06, 2017/09/07-2017/09/17, 2018/08/25-2018/09/05) are not included to avoid the effects of intense storms and initial operation periods of MEPi before May 16, 2017.

**Fig. 2:**
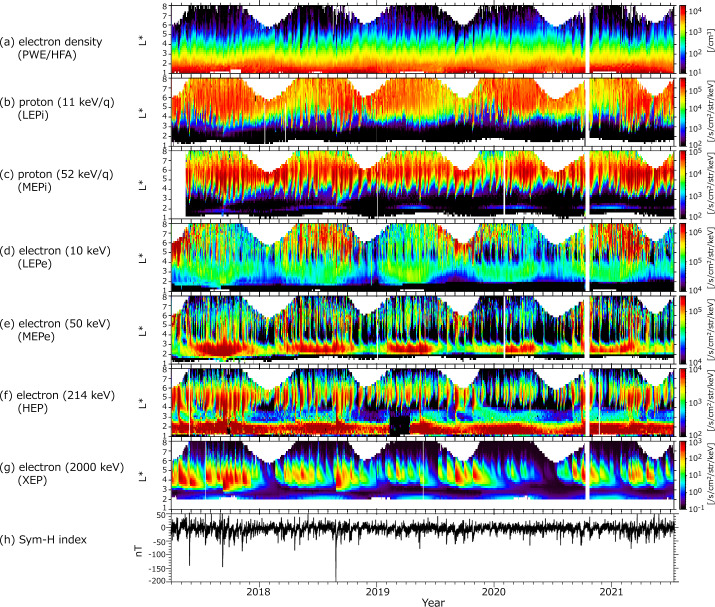
$L$-time diagram of different plasma/particles in the inner magnetosphere obtained from Arase observations. (a) electron density (PWE/HFA), (b) 11 keV/q proton flux (LEPi), (c) 52 keV/q proton flux (MEPi), (d) 10 keV electron flux (LEPe), (e) 50 keV electron flux (MEPe), (f) 214 keV electron flux (HEP), (g) 2000 keV electron flux (XEP), and (h) the Sym-H index. The apogees MLT of Arase are 06:00 (March 2017, May 2018, September 2019), 24:00 (June 2017, October 2018, December 2019), 18:00 (November 2017, February 2019, May 2020) and 12:00 (February 2018, June 2019, September 2020). Note that large flux below $L\sim 2$ at HEP (f) is contamination of the background population

Figure 2 shows the $L$-shell vs time spectrograms of different energy ions, electrons, thermal plasma density, and the Sym-H index (World Data Center from April 2017 to March 2020). During this interval, the apogee magnetic local times (MLTs) of Arase revolved from dawn to midnight, dusk, and noon 3 times. The MLTs at apogee are 06:00 (March 2017, June 2018, September 2019), 24:00 (June 2017, October 2018, December 2019), 18:00 (November 2017, January 2019, May 2020), and 12:00 (February 2018, June 2019, August 2020). Because the magnetic latitude coverage depends on the season, the Arase satellite periodically obtains observations at higher $L$-shell regions. There were several major (minimum Sym-H < −100 nT) as well as moderate magnetic storms during this interval, and large flux enhancements as well as penetration into low $L$-shell regions were observed in the plasma sheet and ring current populations (Figs. 2b–e). Note that the electron flux enhancement measured by LEPe after January 2019 is attributable to a change in the high-voltage level of the micro-channel plates (MCPs). The flux enhancement of 50 keV electrons measured by MEPe at inner $L$-shells appears to be the same as that reported by Ukhorskiy et al. (2014), which shows a zebra-like pattern in its energy-time spectra. During storms, sharp inward depressions in the thermal plasma density, that is, the plasmapause shrinkage, were detected (Fig. 2a). The relativistic electron flux (Fig. 2g) shows different evolution. Large flux enhancements of MeV electrons were observed during the recovery phase of several storms. However, such enhancements are not always associated with storms (Reeves et al. 2003; Miyoshi and Kataoka 2005). Diffusive transport into the lower $L$-shell region was also observed. Sub-relativistic electrons (Fig. 2f, HEP) show similar variations to tens of keV electrons (2e, MEPe), that is, sharp flux enhancements were detected in the storm time. However, flux enhancements continued for a longer time in the sub-relativistic population than in tens of keV electrons. A similar population at slightly inner $L$-shells was measured by both MEPe and HEP, although contamination due to high-energy protons needs to be evaluated for further quantitative analysis.

**Fig. 3:**
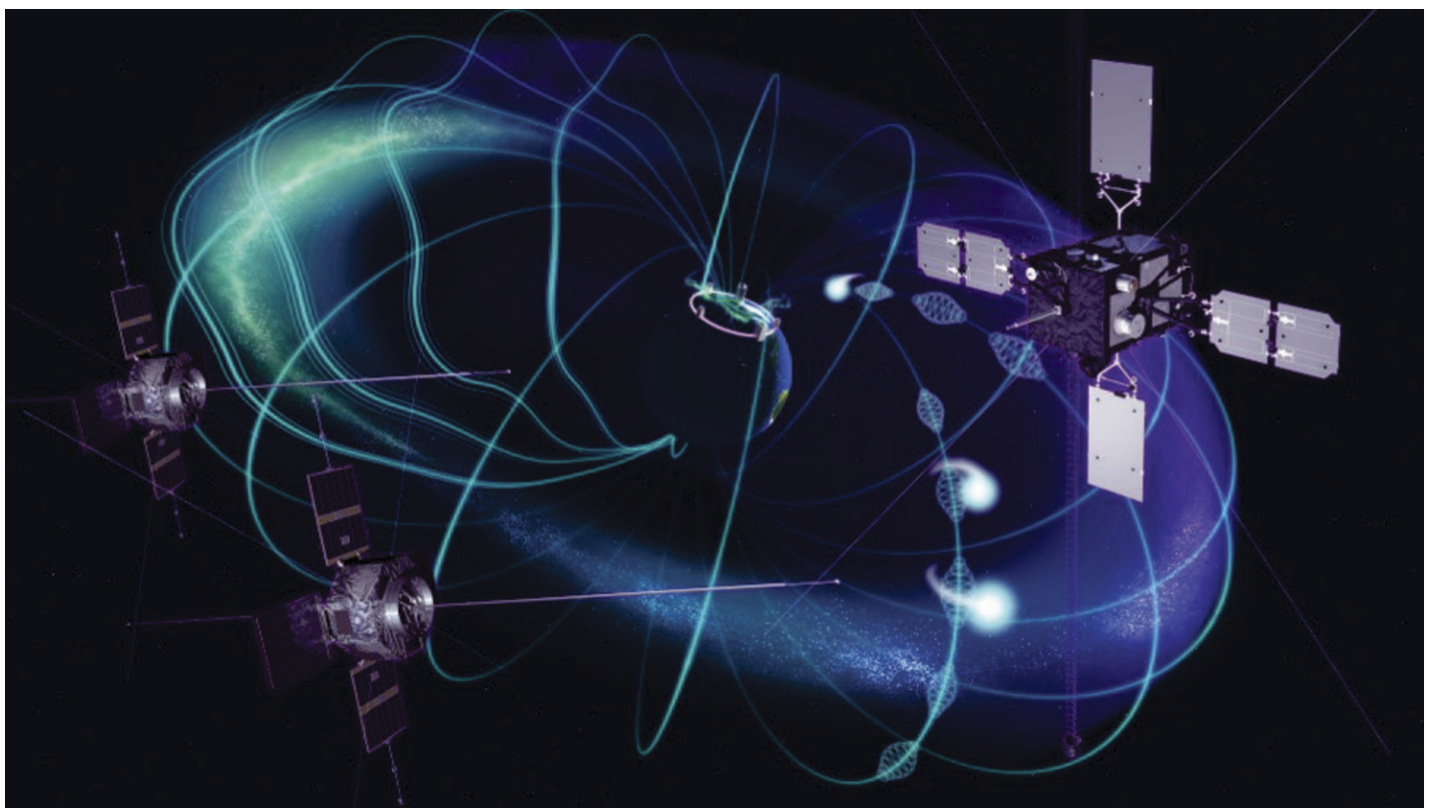
Conceptual drawing of the locations of the Arase and Van Allen Probes satellites in the inner magnetosphere, illustrating how the relative positions of the satellites can be exploited with collaborative observations to expand our knowledge of inner magnetospheric wave and particle dynamics, including the spatial and temporal extent of source regions, wave particle interaction regions, and the relative importance of various acceleration, transport and loss mechanisms at different locations

The launch of Arase provided a unique opportunity for multi-point observations in geospace along with Van Allen Probes as shown in Fig. 3. The Van Allen Probes and Arase orbits have different inclinations. With an inclination of 10°, the Van Allen Probes measured magnetic latitudes within ±21° from the magnetic equator. Meanwhile, Arase has a higher inclination angle of 31° and provides access to higher magnetic latitudes up to 41°. Moreover, the MLT at apogee of the orbit of the Arase precesses faster (270°/year) than that of the Van Allen Probes (200°/year), providing a wide range of local time separations. The details of Van Allen Probes are found in Fox and Burch (2014). The highly complementary orbital configuration of the two missions is ideal for conducting several collaborative scientific campaigns that require coordinated burst measurements. In this paper, we introduce several achievements from the collaborative observations of Arase and Van Allen Probes.

## Scientific Objectives for Cooperative Observations

**Fig. 4:**
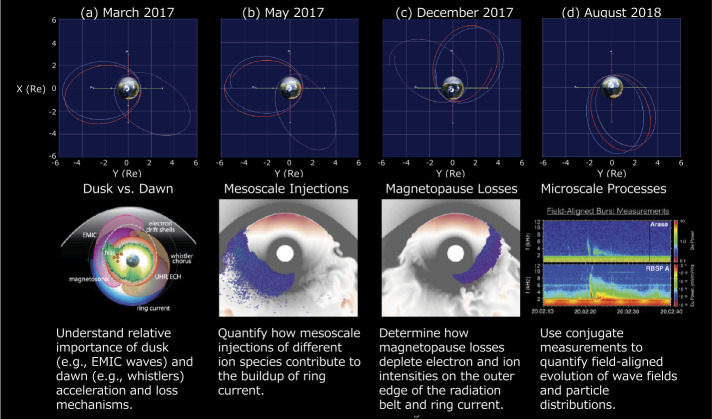
Concepts of collaborative observations of Arase and Van Allen Probes. Arase and Van Allen Probes sometimes observed at different radial distance and local times, while both satellites sometimes observed the same field line at different latitudes

Figure 4 shows examples of the orbits of Van Allen Probes and Arase and the scientific targets identified for each orbital configuration in a white paper that was prepared prior to launch of Arase. The orbit plots indicate that the MLT separation between them changes over time, decreasing from ∼8 h in March 2017 to ∼1 h in August 2018.

In March 2017 (Fig. 4a), the Van Allen Probes were in the dusk/evening sector, and Arase was in the dawn/post-midnight sector. With this orbital configuration, we were able to observe different wave modes at different MLTs. For example, EMIC waves were mainly observed on the dusk side, whereas whistler-mode chorus waves were mainly observed on the dawn side.

In May 2017 (Fig. 4b), the Van Allen Probes were in the dusk sector, and Arase was in the midnight sector. In this case, Van Allen Probes and Arase observed different properties of injections of the plasma sheet electrons and ions, which helped to determine the observation of macroscale injections at different MLTs.

In December 2017 (Fig. 4c), Van Allen Probes were in the pre-noon sector, whereas Arase was in the post-noon sector. During the interval, both satellites may have observed the magnetopause shadowing that occurs in the dayside magnetopause. Here, different particle dynamics at different MLTs help us understand the process of magnetopause shadowing for the loss of the outer portion of the outer belt.

In August 2018 (Fig. 4d), both Van Allen Probes and Arase were in the night sector with a small MLT separation. In this case, the higher inclination of Arase’s orbit allows for the study of the evolution of waves along magnetic field lines.

Both missions prepared a detailed operation plan for the conjugate observations for each phase of orbital configuration. Normal-mode observations were continuously conducted by both missions to acquire data to cover many science topics that require conjugate observations. The satellites observe the wave frequency spectrum DC–400 kHz for Van Allen Probes and DC–10 MHz for Arase in the normal mode. These observations are suitable for overview studies of high-frequency waves, EMIC waves, magnetosonic waves (MSWs), and whistler-mode waves.

Burst-mode operations were also performed. The electric field and wave (EFW) and electric and magnetic field instrument suite and integrated science (EMFISIS) instruments of the Van Allen Probes have several burst modes to measure chorus waves, MSWs, and EMIC waves. The sampling rates of the burst modes of EFW (Wygant et al. 2013) and EMFISIS (Kletzing et al. 2013) are 16.384 and 35 kHz, respectively. The sampling rate of the PWE and waveform capture (WFC) of Arase is 64 kHz (Kasahara et al. 2018a; Matsuda et al. 2018). These data are stored in an onboard data recorder, and the selection and download are performed by examining the data of the frequency–time spectrograms in the normal mode.

## Highlights from Conjugate Observations

As shown in Fig. 4a, Van Allen Probes and Arase performed measurements with varying local time separations, providing an opportunity to specify the region of wave–particle interactions such as interactions between electrons and magnetohydrodynamics (MHD) fast mode waves, interactions with whistler mode, and EMIC waves. In this section, we review selected results from multi-point observations.

### Multi-Point Measurements by the Two Satellites

**Fig. 5:**
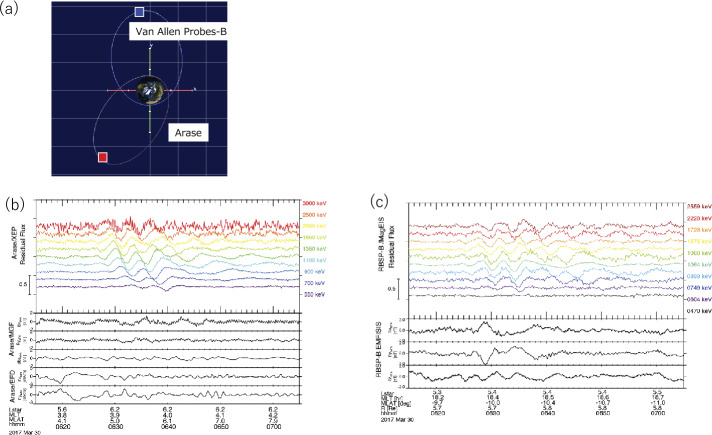
(a) Orbit of Van Allen Probe B and Arase on March 30, 2017, with location at time of observations indicated by rectangles. (b) Residual electron flux from Arase/XEP (top) and magnetic field in the mean field-aligned coordinate system from Arase/MGF and Electric Field Detector (EFD) (bottom). (c) Residual electron flux from MagEIS-B (top) and magnetic field in the mean field-aligned coordinate system from EMFISIS-B magnetometer (bottom; Teramoto et al. 2019)

Teramoto et al. (2019) investigated the resonance between relativistic electrons and Pc5 ultra-low-frequency (ULF) waves on March 30, 2017, using Van Allen Probe B and Arase. Figure 5a shows the orbits of Van Allen Probe B and Arase during this interval. Both satellites were located at $L \sim 6.0$, but the MLTs of (Van Allen) Probe B and Arase were 18.5 and 4.5, respectively, and rectangles indicate the observed locations of both satellites. Figure 5b shows the several-hundred-keV-to-MeV electron flux observed by the XEP onboard Arase and the ambient magnetic fields measured by the Arase magnetic field (MGF) instrument (Matsuoka et al. 2018) between 6:10 and 7:10 UT. Teramoto et al. (2019) used residual flux to emphasize modulations. The residual flux at time t is defined as (J(t) − J_0_)/J_0_, where J(t) is the spin-averaged flux and J_0_ is the running average of J(t) with a time window of 5 min. During this interval, Arase was in the post-midnight sector and periodic modulation of electrons were detected with a period of 170–250 s; however, the satellite did not detect significant magnetic field variations. The modulation of the electrons shows an energy dispersion. The modulation first appears at a higher energy and then lower energy, suggesting that the modulation that causes the periodic variations occurs at different MLTs from the Arase satellite. Figure 5c shows the several-hundred-keV-to-MeV electron flux observed by the Van Allen Probe B magnetic electron ion spectrometer (MagEIS) (Blake et al. 2013) and the ambient magnetic fields measured by the Van Allen Probe B EMFISIS instrument. The same periodic flux modulations with a period of 170–250 s were observed on the dusk side. The ambient magnetic field also shows the same periodic variations, unlike the Arase observations. Thus, modulations between drifting electrons and Pc5 waves occur in a limited MLT region. By combining Pc5 waves observed by the satellites with a ground-based magnetometer network at different MLTs and through a time-of-flight analysis of the energy dispersion of electrons, Teramoto et al. (2019) concluded that drift resonance occurred on the dusk side. Thus, MLT localization is important for understanding the transport of energetic electrons.

**Fig. 6:**
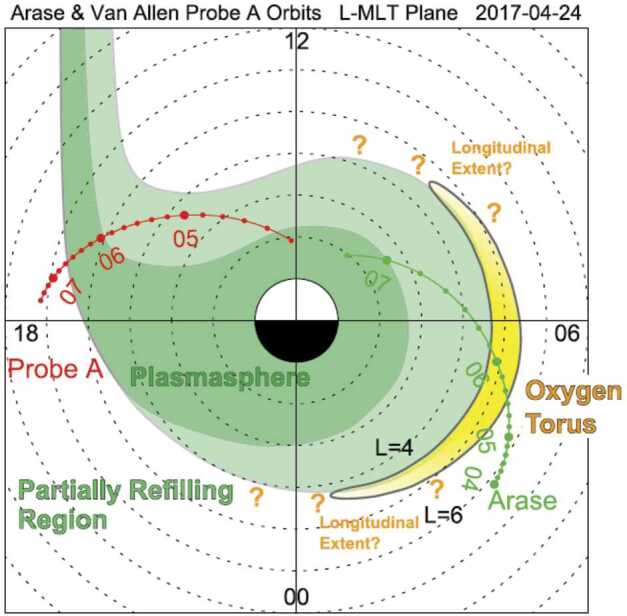
Schematic figure of the structures of the plasmasphere and oxygen torus at 04:00–07:30 UT on April 24, 2017, as inferred from the analysis of Arase and Van Allen Probe A data and the generation scenario. MLT = magnetic local time (Nosé et al. 2018)

Nosé et al. (2018) investigated the longitudinal structure of the oxygen torus on April 24, 2017. During the time interval selected, Arase moved from $L = 6.2$ to 2.0 in the morning sector, whereas Probe A moved from $L = 2.0$ to 6.2 in the afternoon sector. Nosé et al. estimated the mass density from the frequency of ULF pulsations detected by Van Allen Probes/EMFISIS and Arase/MGF as well as the local electron density measured by Van Allen Probes/EMFISIS and Arase/PWE and HFA. In this event, Arase detected an enhancement of the average plasma mass up to ∼3.5 amu around $L$ = 4.9–5.2 in the dawn sector, indicating that the plasma consists of ∼15% O^+^ ions. On the other hand, Probe A showed no clear enhancements in the average plasma mass in the dusk sector. Figure 6 presents a summary of events in the Van Allen Probe A and Arase. Nosé et al. (2018) claimed that the oxygen torus does not extend over all MLTs but is skewed toward the dawn and suggested that the torus is crescent-shaped or pinched. A similar structure of the oxygen torus was also confirmed by the simultaneous observations of Arase and Van Allen Probes A and B for the September 12, 2017, event (Nosé et al. 2020).

**Fig. 7:**
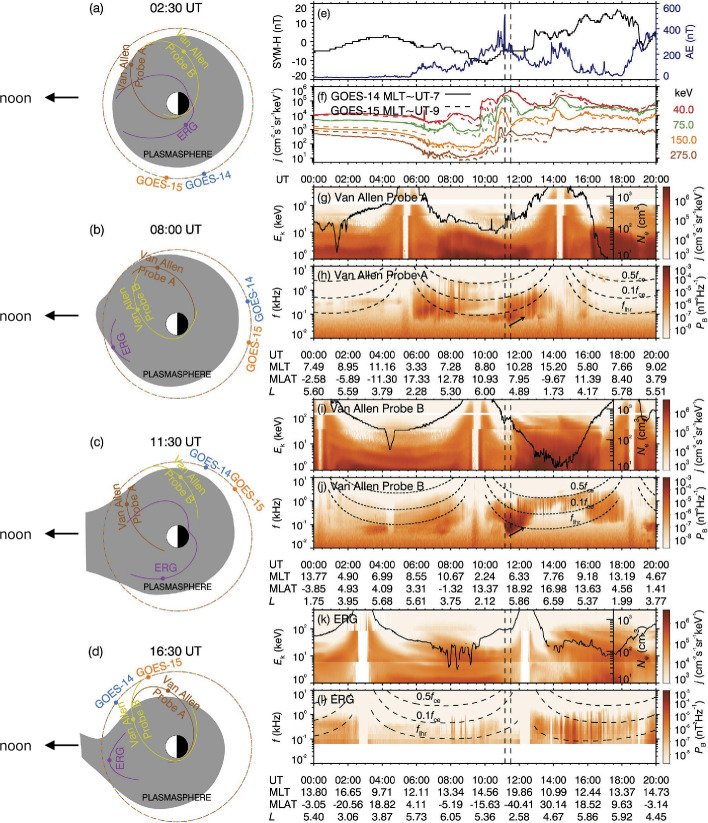
(a–d) Overview of the 13 January 2018 plasmaspheric hiss event. Schematic diagrams of plasmasphere structures (shadows) and spacecraft locations (colored dots) at 02:30, 08:00, 11:30, and 16:40 UT, respectively. The plasmasphere shadows are illustrative cartoons based on the density profiles of the Van Allen Probes and Arase missions, whose trajectories (solid lines) covered the period 00:00–05:30, 05:30–10:00, 10:00–14:00, and 14:00–20:00 UT in a–d, respectively. (e) Geomagnetic activity indices SYM-H and AE. (f) Hot electron differential flux profiles. (g, i, k) Hot electron differential energy spectra, with overplotted local electron densities. (h, j, l) Wave magnetic power spectra, with the overplotted frequencies 0.5 fce (equatorial electron cyclotron frequency), 0.1 fce, and f_LHR_ (equatorial lower hybrid resonance frequency). The vertical dashed lines mark the sudden enhancement of hiss waves and the substorm injection front observed by Van Allen Probe B, respectively. The blank region represents the data gap (Liu et al. 2020)

Multi-point observations are useful for identifying the distributions of injected particles and resultant wave activities. Liu et al. (2020) analyzed a substorm event on January 13, 2018, when the apogee of Van Allen Probes A and B was at pre-noon, whereas that of Arase was at post-noon. Figure 7 shows the locations of the satellites, and energetic electrons and frequency spectrum data observed by Van Allen Probes A and B and Arase. Figure 7 (A)-(D) show the locations of Arase and Van Allen Probes A and B as well as GOES-14 and 15 satellites. The shaded region is a schematic figure of the plasmasphere. Panel (E) shows the SYM-H and AE index. Panel (F) shows that both GOES-14 and GOES-15 observed injections of tens to multi-hundred keV electrons in the post-midnight region. The weak substorms injected hot electrons (panels G, I and K) up to $L = 5.5$, according to the Van Allen Probe A/Helium, Oxygen, Proton, and Electron (HOPE) (Funsten et al. 2013) and MagEIS observations in the midnight–dawn–noon sector. The enhanced plasmaspheric hiss (panels H, J and L) exhibited a strong asymmetry in MLT. The plasmaspheric hiss power peaked on the dawn side (Van Allen Probe A/EMFISIS), followed by an order-of-magnitude decrease on the noon side (Van Allen Probe B/EMFISIS), and a near disappearance on the afternoon side (Arase/PWE/onboard frequency analyzer (OFA). After or at the onset of substorm around 11:00 UT, Van Allen Probe B detected the enhancement of hot electrons (panel G) and hiss waves (H) near dawn. Van Allen Probe A detected hiss waves (panel J) with lower intensities in the absence of electron injection (I) close to noon, and Arase/low-energy particle experiment (LEPe), medium-energy particle experiment (MEPe), and HEP observed neither electron injection (panel K) nor hiss waves (L) inside the plasmasphere on the dusk side. The plasmaspheric hiss observed by Van Allen Probes A and B had frequencies drifting slowly with time; their lower cutoff increased from ∼30 to ∼100 Hz on a timescale of 1 h. After 14:00 UT, the plasmasphere was substantially eroded. In the noon-side plasmaspheric plume, Arase detected the injection of hot electrons and enhanced hiss waves. In the pre-noon sector, there were moderate plasmaspheric hiss waves (Van Allen Probe B) but intermittent and weak chorus waves (Van Allen Probe A). These multi-point measurements identified enhancements of the hiss waves associated with injections and propagation into different MLTs as well as subsequent damping.

### Observations of Plasma Waves

**Fig. 8:**
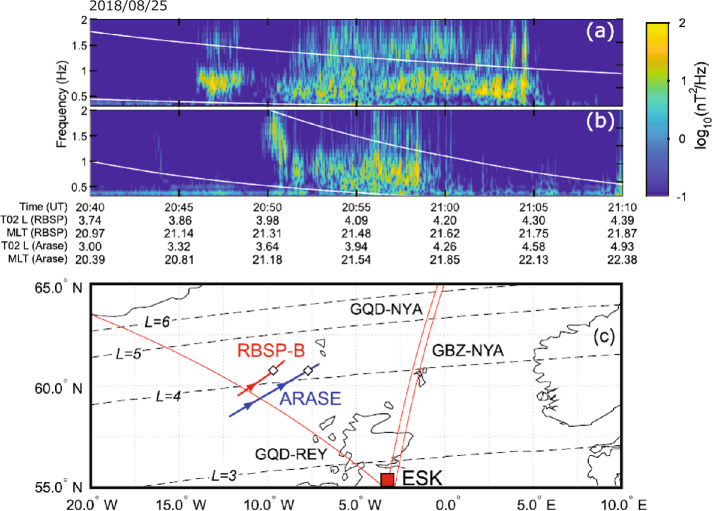
(a) Spectrogram of the RBSP-B perpendicular wave power (field-aligned coordinates), with the helium and oxygen gyrofrequencies plotted in white, on August 25, 2018. (b) Same as (a) but for the data from Arase’s MGF instrument. (c) Map of the event region with the RBSP-B and Arase T02 footprints in red and blue, respectively (arrows indicate the direction of travel, while white diamonds indicate the point of the closest approach); three AARDDVARK VLF paths in red; and the Eskdalemuir search-coil magnetometer in a red square. $L$-shells from 3–6 are shown as dashed black lines (Hendry et al. 2020)

Hendry et al. (2020) identified the spatial distribution of the source region of EMIC waves and energetic electron precipitation through conjugate observations of EMIC waves by the Van Allen Probe B and Arase and ground-based observations on August 25, 2018. During the observations, both satellites were at almost the same MLT and $L$-shell and different latitudes. Van Allen Probe B was near the magnetic equator, while Arase was at high latitudes of approximately −30°. Figure 8a and b show the frequency–time spectrograms of EMIC waves from the Van Allen Probes/EMFSIS and Arase/MGF, respectively. The Van Allen Probes detected fine structure elements of EMIC waves (Matsuda et al. 2018), implying a rising tone. Arase was located much further south along the field line at a magnetic latitude of approximately −30°, likely beyond the EMIC source region. As shown in Fig. 8c, the satellite footprints were separated by ∼800 km. These observations are of the same wave event, with strong similarities in the MLT locations of the wave observations. Using the ground-based low frequency (LF) radio wave network and conjugate observations from Van Allen Probe B and Arase, Hendry et al. (2020) identified that the source region of EMIC waves and energetic electron precipitation for this event are distributed around 11° longitude. Miyoshi et al. (2019) also investigated EMIC waves using Van Allen Probes and Arase and discovered a new source of plasmaspheric EMIC waves. Mode conversion of MSWs to EMIC waves occurred with M/Q = 2 ions if the MSWs propagated into low $L$-shells ($L < 2$).

**Fig. 9:**
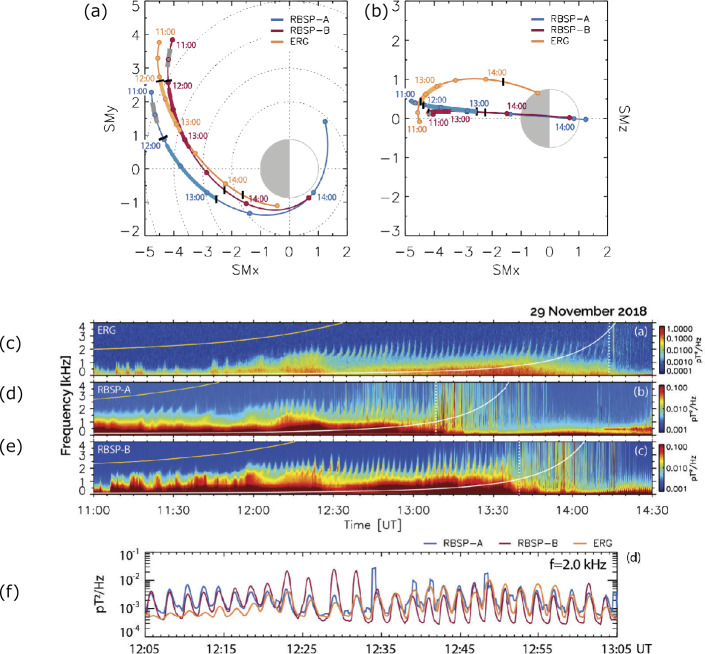
(a)(b) Position of Arase (orange) and Van Allen Probes A (blue) and B (red) from 11:00 to 14:30 UT on November 29, 2018, in solar magnetic coordinates. Solid colors represent the trajectory of the satellites with overlapped solid gray lines indicating the first and main conjugated events. Each dot represents 30 min. Vertical black solid bars indicate timings where each satellite observed QP emissions starting at 12:06 UT. (c) (d) (e) Power spectral density for Arase and Van Allen Probes A and B between 11:00 and 14:00 UT (0–4 kHz). The bottom panel shows (f) the intensity variations (smoothed with a 30 s window) as a function of time for $f = 2$ kHz for Arase (orange) and Van Allen Probes A (blue) and B (red) (Martinez-Calderon et al. 2020)

Martinez-Calderon et al. (2020) investigated the spatial extent of quasi-periodic (QP) emissions in the Very Low Frequency (VLF) range on November 29, 2018, using Van Allen Probes EMFISIS and Arase PWE/OFA. During the interval, three satellites moved from the pre-midnight to the morning sector, as shown in Figs. 9a and b. Data from the three satellites show one-to-one correspondence of QP emissions and reveal the spatial extent as 1.2 Re in terms of radius and 2.2 h in MLT. During the event, the satellites also observed the frequency sweep rate of the QP elements associated with magnetic disturbances. Martinez-Calderon et al. (2020) discussed the possibility of changes in the source mechanisms. These observations distinguish the spatial and temporal variations of the QP emission and are important for understanding the spatial extent of the waves.

**Fig. 10:**
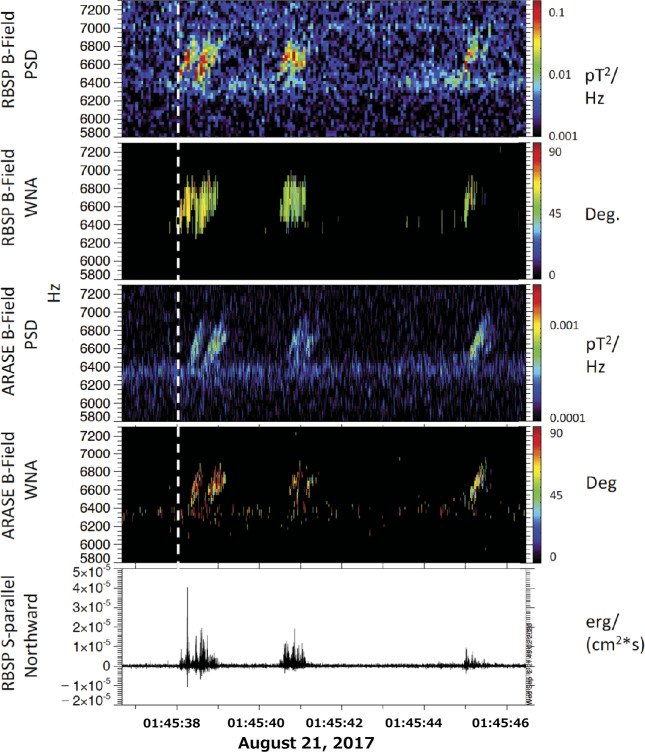
5.8-7.3 kHz burst magnetic field spectrograms from (a) Van Allen Probes A EFW, (c) Arase PWE/WFC from 01:45:36-01:45:46 UTC on August 21, 2017. (b) Van Allen Probes and (d) Arase wave normal angles of the magnetic field waveform data from 0 deg to 90 deg, and (e) parallel Poynting flux of the waves observed on Van Allen Probes A with positive = northward (Colpitts et al. 2020)

Colpitts et al. (2020) presented the first direct observations of the same rising tone chorus elements propagating from the equatorial plane measured by Van Allen Probes to off-equatorial plane measured by Arase on August 21, 2017. The rising tone element is observed first on Van Allen Probes and becomes more oblique and significantly attenuated on Arase. Figure 10 shows 5.8 – 7.3 kHz burst magnetic field spectrogram from (a) EFW of Van Allen Probes A, and (c) WFC of Arase. Figure 10 (b) and (d) indicate wave normal angles observed on Van Allen Probes A, and Arase, respectively. Figure 10 (e) shows the parallel Poynting flux of the waves by Van Allen Probes-A, and positive means the northward propagating waves. Colpitts et al. (2020) compared these observations with a ray-tracing analysis, and they confirm that the rising tone elements are generated parallel to the ambient magnetic field near the equator, and then propagate through the medium unducted to Van Allen Probes and then to Arase with the observed time delay.

### Observations of the Rapid Flux Decrease of the Outer Belt

**Fig. 11:**
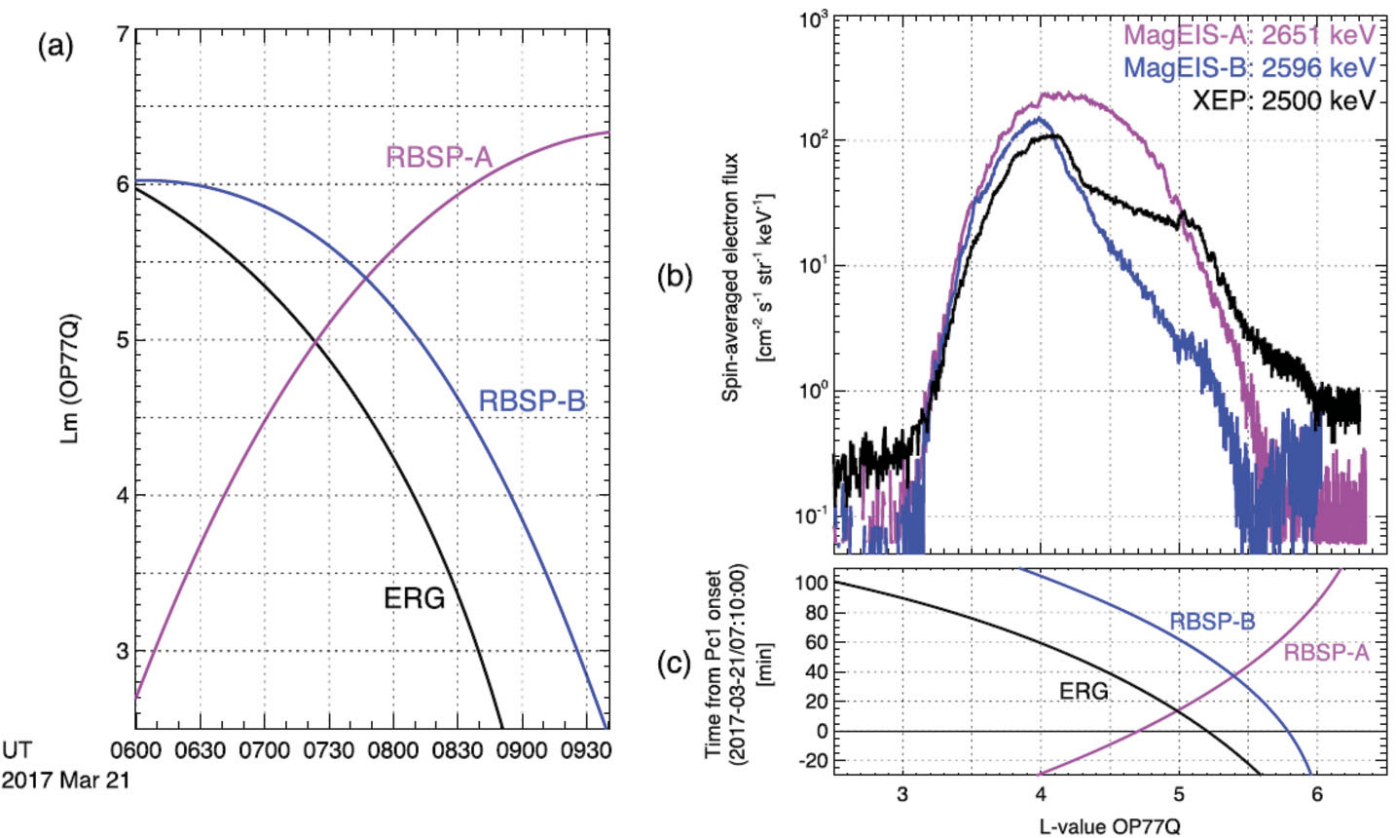
(a) Location of Van Allen Probe-A (magenta), Van Allen Probe-B (blue), and Arase (black) around the time of interest. (b) Spin-averaged fluxes of ∼2.5-MeV electrons observed by Van Allen Probe-A/MagEIS (magenta), Van Allen Probe-B/MagEIS (blue), and Arase/XEP (black) as a function of $L$-value. (c) Time after the Pc1 wave onset at ATH (0710 UT on 21 March) as a function of location of Van Allen Probe-A (magenta), Van Allen Probe-B (blue), and Arase (black) in $L$. The $L$-shell of this figure is the McIlwain $L$-shell derived from Olson and Pfitzer 1977 (Olson and Pfitzer 1977) model (Kurita et al. 2018)

Kurita et al. (2018) identified relativistic electron precipitation based on EMIC waves observed on March 21, 2017. Similar to Teramoto et al. (2019) (Fig. 5), the apogee lines of Van Allen Probes A and B were at pre-midnight, whereas that of Arase was at post-midnight. As shown in Fig. 11a, Van Allen Probes and Arase traversed the outer radiation belt during the activation of the EMIC waves, with Arase and Van Allen Probe B on an inbound pass and Van Allen Probe A on an outbound pass during the EMIC wave event. The Arase EMIC wave observation was followed by the Van Allen Probe B observation, with a time difference of ∼50 min. Figure 11b shows the $L$-shell profiles of ∼2.5 MeV electron fluxes measured by Arase/XEP (black) and Van Allen Probes A (magenta) and B (blue)/MagEIS. Figure 11c shows the relationship between the satellite $L$-shell locations and time after the EMIC wave onset determined by the induction magnetometer networks of the ERG ground-based observation team (Shiokawa et al. 2017). The Van Allen Probe A observation showed a smooth $L$-shell profile of ∼2.5 MeV electron fluxes throughout the outer radiation belt. The $L$-shell profile measured by XEP was significantly deformed compared to that observed by Van Allen Probe A in the $L$-shell range of 4.2–4.9. This $L$-shell range of flux depletion roughly corresponds to the region of enhanced EMIC wave activity. Following the inbound pass of Arase, Van Allen Probe B observed a further decrease in ∼2.5 MeV electron fluxes. Here, Arase and Van Allen Probes showed that ∼2.5 MeV electron fluxes substantially decreased at $L > 4.2$, within 1.5 h, in association with the enhanced EMIC wave activity. Using multi-point observations, rapid depletion of the outer belt electrons was identified. These observations are important for specifying the possible time scale and region of pitch-angle scattering by wave–particle interactions.

## Intercalibration Between Arase and Van Allen Probes

### Intercalibration of Plasma/Particle Observations

**Fig. 12:**
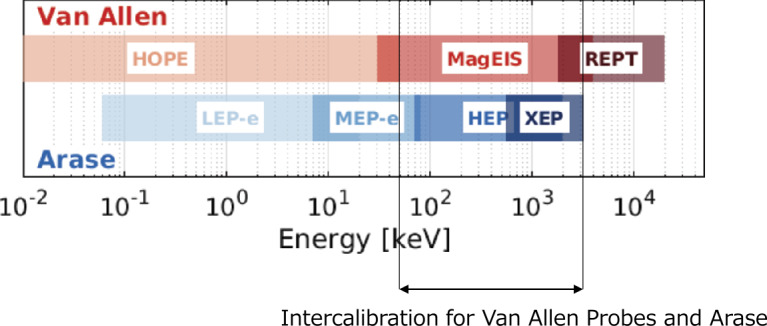
Electron energy range coverage for the particle instruments of Arase and Van Allen Probes (Indicate the energy range of interest for calibration)

In this section, we describe the intercalibration between the Arase and Van Allen Probes energetic electron detectors, specifically the MEPe, HEP, and XEP instruments onboard Arase and the relativistic electron proton telescope (REPT) (Baker et al. 2012) and MagEIS instruments onboard Van Allen Probes. Figure 12 shows a comparison of the approximate energy coverage of these instruments. We focus on measurements in the ∼50 keV to ∼3 MeV energy range for the inter calibration in this paper.

**Fig. 13:**
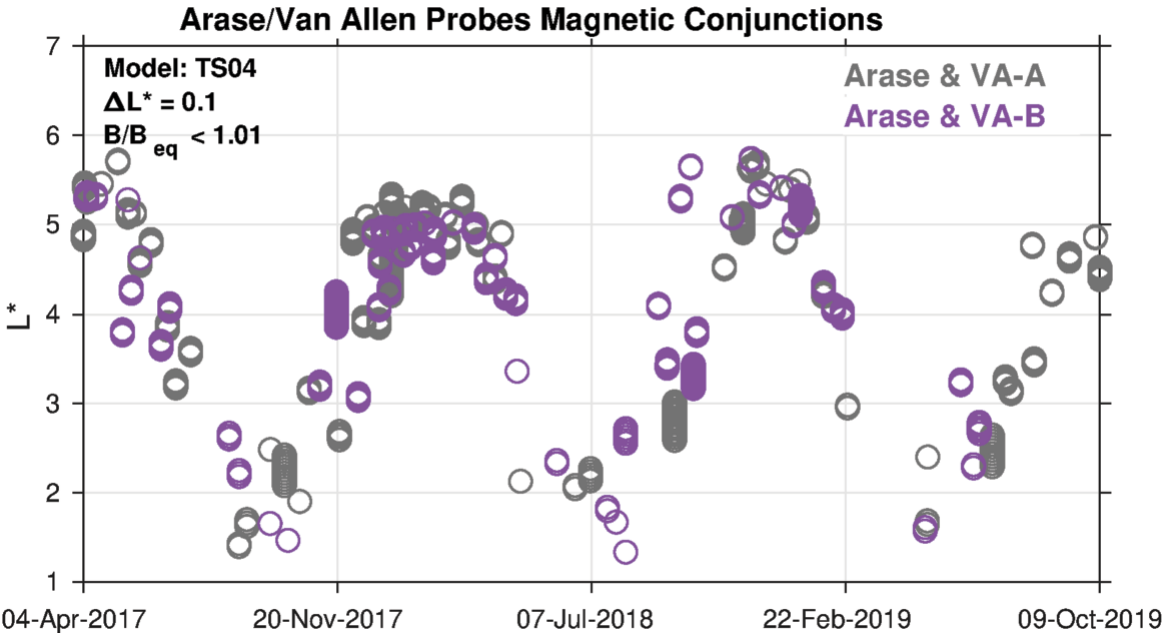
Magnetic conjunctions between Arase and Van Allen Probes. The time interval displayed is that common to both missions. The conjunction criteria are that the two spacecrafts are within 0.1 $L^{*}$ of one another and near the magnetic equator ($B/B_{eq} < 1.01$)

It is important to compare measurements from the different spacecraft at similar magnetic latitudes because the electron angular distributions are generally strongly peaked near the magnetic equator, and we compared the spin-averaged flux. Figure 13 shows “magnetic” conjunctions between Arase and each of the two Van Allen Probes when the two spacecraft were within 0.1 $L^{*}$ of one another and near the magnetic equator, as measured by the ratio of the local magnetic field at the spacecraft (B) to the equatorial field strength along the same field line (B_eq_). These magnetic ephemerides were computed using the TS04D magnetic field model (Tsyganenko and Sitnov 2005). Thus, during a magnetic conjunction, both spacecraft observe roughly the same portion of the electron angular distribution at the same $L^{*}$-shell, although they may be separated in terms of MLT. At higher energies (∼1 MeV), electrons drift rapidly (∼5 min) through all MLTs; thus, the distributions during magnetic conjunctions are expected to be similar, which can help reveal offsets between instruments. However, at lower energies (∼ <100 keV), the drift periods are longer, and the flux distributions also exhibit certain asymmetry in terms of MLT, leading to physical differences that are unrelated to intercalibration.

**Fig. 14:**
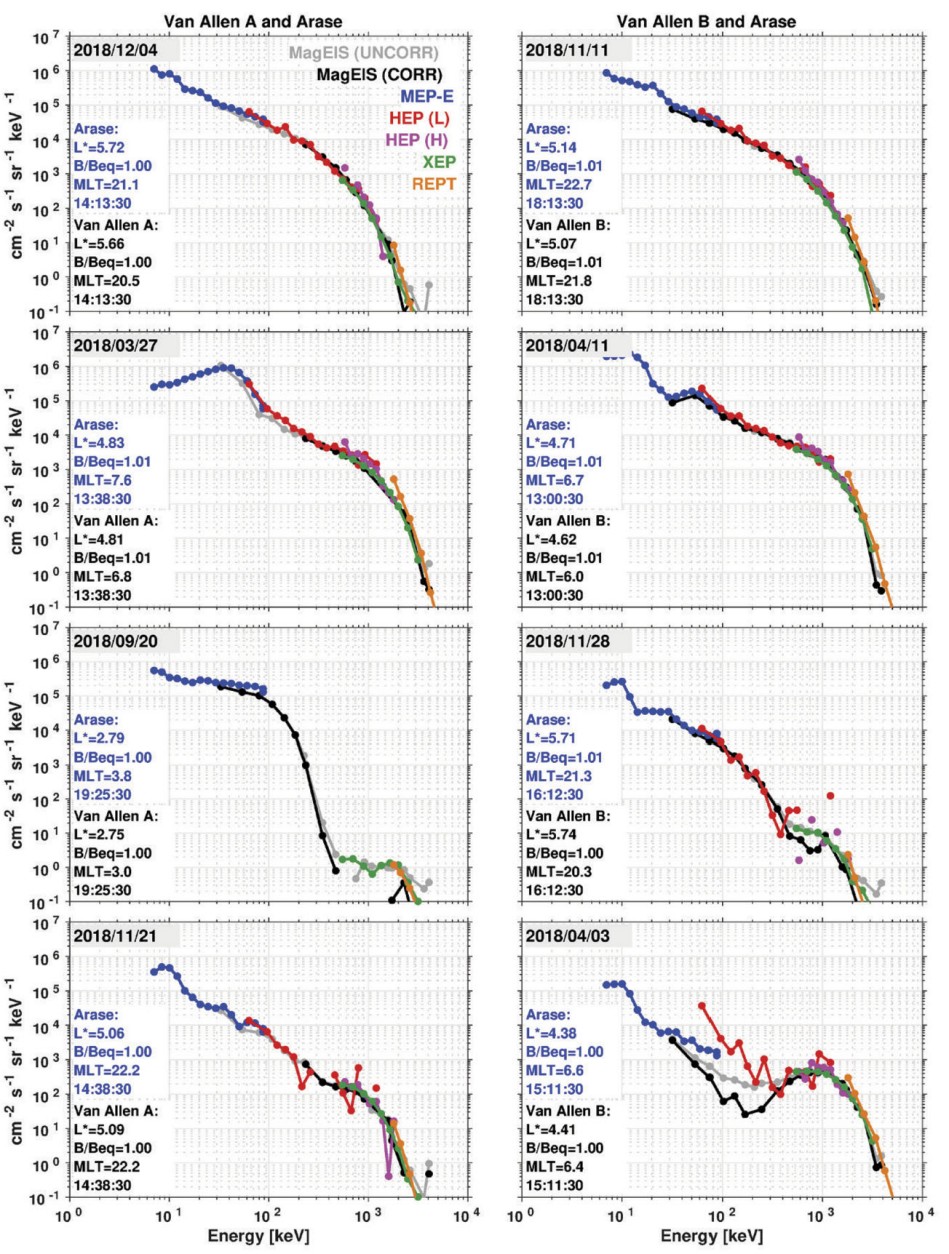
Differential, spin-averaged electron flux spectra from Arase and Van Allen Probe A (left column) and B (right column). Each panel shows the spectra during physical conjunctions, where the two spacecrafts were close with $L^{*}$ (<0.1), MLT (<1 h), and near the magnetic equator ($B/B_{eq} < 1.01$). Both uncorrected and background corrected MagEIS data are shown

Figure 14 shows examples of electron differential flux spectra from Arase (MEP-e, HEP, and XEP) and Van Allen Probes (MagEIS and REPT). These correspond to a subset of magnetic conjunction events shown in Fig. 13 that satisfy the condition of a small spacecraft MLT separation of less than 1 h. In Fig. 14, we see good agreement between the Arase and Van Allen Probe electron measurements across a wide range of energies. We note that both the intensity and the shape of the spectra are consistent between the measurements from the two missions. There are notable differences, however, particularly when the foreground fluxes are low. For example, in the panel for Probe B and Arase (right column) on 2018/11/28, we see a significant amount of scatter in the fluxes near 500 keV – 1 MeV. The flux levels are low at this time (∼10^1^ cm^−2^ s^−1^ sr^−1^ keV^−1^) and thus subject considerable Poisson/counting error relative to other time intervals when the count rates/fluxes are higher (e.g., 2018/11/11 when the flux levels are ∼10^2^-10^3^ cm^−2^ s^−1^ sr^−1^ keV^−1^ in this same energy range). An additional source of measurement error occurs when multi-MeV electron flux is elevated, which can produce bremsstrahlung and penetrating contamination in the measurements. This effect is most evident when foreground fluxes are low. For example, in the bottom right panel, the significant differences between the uncorrected and background-corrected MagEIS measurements indicate that there is substantial contamination from multi-MeV electrons in the ∼100–300 keV energy range, where the foreground fluxes are low. Thus, there is a significant error in the measurements at this time, due to both Poisson noise (low counting statistics) and background contamination. Note that background counts caused by protons and bremsstrahlung X-ray are not taken into consideration in the current version of the HEP data. Therefore, in terms of making the most meaningful comparisons, we compare the HEP data with uncorrected MagEIS data.

**Fig. 15:**
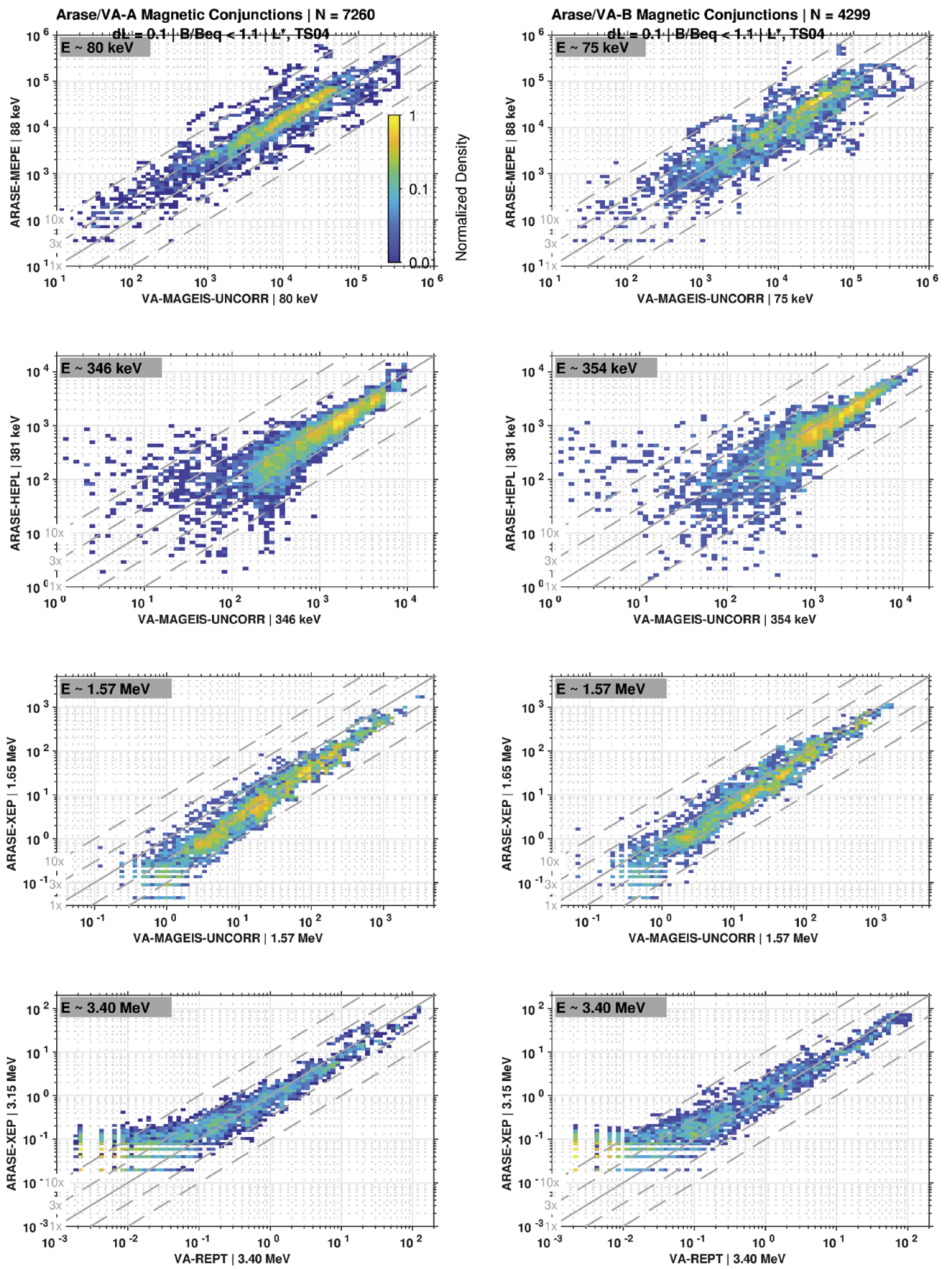
Scatter plot comparisons of electron flux for similar energy channels on Arase and Van Allen Probe A (left column) and B (right column) during magnetic conjunctions. Each panel shows Van Allen Probe flux on the horizontal scale and Arase flux on the vertical. All flux units are (cm^2^ s sr keV)^−1^. Guidelines are shown indicating factors of 1x, 3x, and 10x. The color scale is normalized to the maximum occurrence frequency over all flux bins

As an additional intercalibration procedure, Fig. 15 shows scatter plot comparisons between similar energy channels from two instruments, one from Arase and one from the Van Allen Probes. Guidelines are shown indicating factors of 1x, 3x, and 10x. In these comparisons, all magnetic conjunctions are shown; to increase the number of events, the equatorial criterion was relaxed to $B/B_{eq} < 1.1$, and there was no requirement for proximity in terms of MLT. In each panel, the spin-averaged electron flux from Van Allen Probes is shown on the horizontal axis, while flux from Arase is shown on the vertical axis. The specific instrument that provides the energy channel is indicated by the axis labels. Here, we observe good agreement between the electron measurements from the two missions, exhibiting approximate linearity with intensity. Overall, the fluxes also show agreement with one another to within a factor of ∼3 for most magnetic conjunctions, although some outliers are noted. In the second row, we observe a more pronounced disagreement between HEP and MagEIS at lower flux levels. Again, this is likely due to the combined effects of low foreground levels in the presence of multi-MeV electrons that can produce errors in the measurements.

Other studies about inter calibrations between Van Allen Probes and Arase are reported using the long-term observations of both satellites (Sandberg et al. 2021; Szabo-Roberts et al. 2021). Such inter-calibrations are important for quantitative comparisons with the same qualities.

### Intercalibration of Plasma Wave Observations

Intercalibration of plasma wave experiments on Van Allen Probes (EMFISIS) and Arase (PWE) becomes possible during intervals when these spacecrafts are in proximity to each other. However, because the spacecraft separation is not sufficiently small to be comparable to the wavelength (less than a few tens of kilometers) of the waves of interest, we cannot expect an exact match of waveforms between the two spacecraft. The cross-calibration of wave instruments can be made only when we are reasonably certain that the two spacecraft observe waves originating from the same source and that these waves propagate at the two spacecraft locations with similar intensity and polarization.

**Fig. 16:**
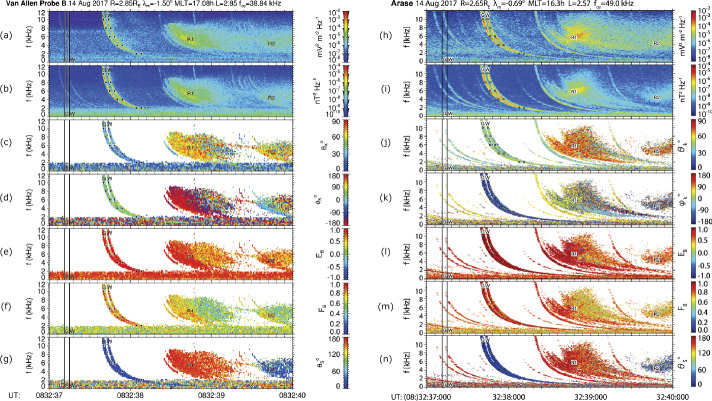
Results of the polarization and propagation analysis of electric and magnetic field measurements recorded by the EMFISIS instrument onboard the Van Allen Probe B (a-g) and PWE instrument onboard Arase (h-n) in the frequency band below 12 kHz on 14 August 2017 during 3 s from 08:32:37 UT. (a)(h) Sum of the power spectral densities from two spin-plane electric field antennas; (b)(i) Sum of the power spectral densities from three orthogonal magnetic field antennas; (c)(j) Angle between the background magnetic field line and the wave vector obtained from the SVD analysis of the magnetic spectral matrix; (d)(k) Azimuth of the wave vector in the plane perpendicular to the background magnetic field line measured from the outward direction and increasing eastward; (e)(l) Ellipticity and (f)(m) planarity of the magnetic field polarization obtained from the SVD analysis of the magnetic spectral matrix; and (g)(n) Angle between the background magnetic field line and a spectral estimate of the Poynting vector. Black vertical lines show the time of two lightning return strokes as detected by the GLD300 (G) WWLLN (W), respectively. The results of ray-tracing analysis of the two corresponding whistlers are shown by black squares at frequencies of 2, 4, 6, 8, and 10 kHz. Two time periods of broadband emissions assumed to be magnetospheric reflections (R1 and R2) are also marked (Santolík et al. 2021)

Figure 16 shows results of an analysis of Van Allen Probe B and Arase measurements during an event fulfilling the above conditions and discussed in detail by Santolík et al. (2021). On August 14, 2017, at 08:32:37 UT, the two spacecraft were in the vicinity of the geomagnetic equator, with a small separation and magnetic latitudes of −1.5° and −0.7°, radial distances of 2.85 and 2.65 Earth radii, and MLTs of 17.1 and 16.3 h, respectively for Van Allen Probe B and Arase. At 600 ms after 08:32:37.600 UT, both spacecraft recorded a pair of intense whistlers, nearly at the same time and with the same dispersion. The time interval between the two whistlers was ∼60 ms, which was the same for both spacecraft, and the waves were observed with approximately the same intensity of electromagnetic field components (Fig. 16a, b and h, i). It is therefore highly likely that both observed electromagnetic waves originated from the same sources.

A remarkably similar dispersion at the two spacecraft indicates that both spacecraft might have observed whistlers propagated through the same duct with an increased plasma density. This can be verified by analyzing the wave vector directions using the singular value decomposition (SVD) of the magnetic spectral matrix (Santolik et al. 2003) as shown in Fig. 16c, d and j, k. At Van Allen Probe B, the wave normal angle $\theta _{k}$ is found to be directed 15–30° from the local background field line (panel c) and away from the Earth. At Arase, the wave vectors are directed 35–50° from the field line and toward the Earth (panel j). These angles are always lower than the Gendrin angle; therefore, the whistlers propagate inward or outward according to the analysis results for the wave vectors, which are different for the two spacecraft. As Arase is, during these measurements, closer to the Earth than Van Allen Probe B, the duct is most likely aligned to a magnetic field line lying somewhere between the two spacecraft. Thus, the observed waves stem from this duct to both spacecraft, inward to Arase, and outward to Van Allen Probe B.

Figure 16e and l confirm that the polarization of these waves is right-handed and nearly circular (Santolik et al. 2002), while Figs. 16f and m, based on the SVD method, indicate that the magnetic field polarization is well concentrated into a single plane on both spacecraft. Spectral estimates of the Poynting vector (Santolík et al. 2010) in Figs. 16g and n show that the two whistlers arrive at both spacecraft from the southern hemisphere. The subsequent intensification of broadband noise after 08:32:38.400 UT, marked as R1, and propagating unducted with highly oblique wave vectors, originated from the north. Similar noise after 08:32:39.600 UT (marked as R2) propagated from the south. These intervals of broadband noise show similar polarization and propagation properties on both spacecraft, and likely represent magnetospheric reflections and/or triggered hiss linked to the observed whistlers.

Source lightning discharges for these two whistlers were found in the Indian Ocean, ∼1000 km southwest of Adelaide, Australia. First, the global lightning dataset GLD360 detection network (Said et al. 2010; Rudlosky et al. 2017) identified a strong negative return stroke with a peak current of −134 kA at geographical coordinates of 40.5^o^S and 130.6^o^E, occurring at 08:32:37.188 UT. After 60 ms, at 08:32:37.248 UT, the worldwide lightning location network (WWLLN) detected another strong discharge with an equivalent peak current of 205 kA (Hutchins et al. 2012a,b) at 40.4^o^S and 130.6^o^E. These lightning positions were in proximity to the magnetic footprints of Arase and Van Allen Probe B at that time, which were at 39.0^o^S and 119.4^o^E and 40.9^o^S and 128.7^o^E, respectively.

The correspondence between the discharges and whistler traces can be verified by analyzing the group delays at different frequencies. As the electron cyclotron frequency $f$_ce_ measured by the EMFISIS magnetometer at Van Allen Probe B was 38.8 kHz, the whistlers were observed over a significant fraction of the frequency band below $f$_ce_. Therefore, the simple whistler dispersion approximation for frequencies much lower than the local group velocity maximum at $f$_ce_/4 becomes invalid, and a more complex approach is necessary. We performed a backward ray-tracing simulation with an adaptive integration step (Cerisier 1970; Santolik et al. 2009), beginning from the position of Van Allen Probe B, and using a diffusive equilibrium model of the plasma density distribution. We obtained the group delays between the spacecraft position and the ionosphere at five frequencies between 2 and 10 kHz, using an initial wave vector inclined 20° inward from the local field line, antiparallel to the direction obtained from the Van Allen Probe B measurements. A field-aligned duct at $L = 2.8$, between the two spacecraft, with a small 5% increase in the plasma density over a width of 0.1 Earth radii was sufficient for guiding the waves down to the ionosphere. With a model temperature of 1000 K, the plasma density had to be decreased to 70% relative to the value of 1800 cm^−3^ obtained at Van Allen Probe B from local measurements of the upper hybrid resonance (Kurth et al. 2015), to account for inaccuracies in the model of the unknown density distribution along the duct. The results for the two source return strokes are shown as black boxes in Fig. 16, and match well with the observed whistlers (Figs. 16a and b). The same duct was used for a similar backward ray-tracing simulation from the Arase position, with an initial wave vector 40° outward from the field line, antiparallel to the direction obtained from the Arase data. The duct successfully guided the waves to the ionosphere. The best fit was obtained for 44% of the measured equatorial density of 3600 cm^−3^ owing to the inaccuracies of our density distribution model. The simulated group delays then correspond well with the observed whistlers (Figs. 16h and i).

**Fig. 17:**
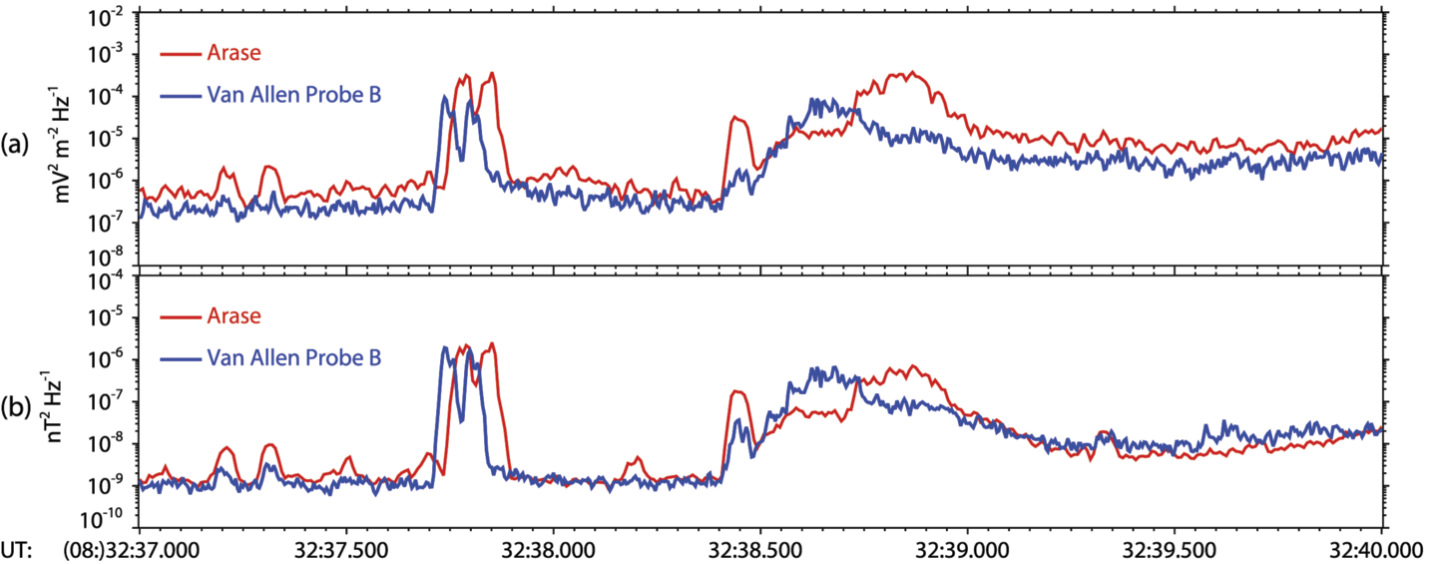
Average power spectral density calculated in a 1 kHz band around 6 kHz for the time intervals in Fig. 13. (a) Sum of the power spectral densities from two spin-plane electric field antennas: (b) Sum of the power spectral densities from three orthogonal magnetic field antennas. Red line: Arase PWE measurements; Blue line: Van Allen Probe B EMFISIS measurements (Santolík et al. 2021)

Therefore, we not only verified that the sources of the observed whistlers correspond to the lightning return strokes detected by two different ground-based networks but also independently confirmed that the absolute time tagging of wave measurements on both spacecraft is correct within an uncertainty of less than 10 ms. The polarization and propagation analyses of the two-spacecraft described above also provide consistent results. Figure 17a, however, shows that the currently archived electric field intensities at the two spacecraft do not match perfectly: Arase detects approximately four times larger power spectral densities of the observed whistlers (corresponding to twice the electric field amplitudes) as compared to measurements of Van Allen Probe B. This demonstrates that it is necessary to consider the antenna–plasma interactions (Hartley et al. 2017; Matsuda et al. 2021). At 6 kHz, the Van Allen Probes spin plane antennas give 84% of the correct electric field amplitude for the measured plasma density, and the Arase electric antennas yield 130% of the correct value. These corrections bring the observed amplitudes within a 33% uncertainty which can be linked to differences in the directions of the measured spin-plane components on the two spacecraft. Figure 17b shows that in the case of triaxial magnetic field measurements of the two identified strong whistlers the values obtained by the two-spacecraft match within an ∼30% uncertainty interval of the magnetic field power spectral densities, corresponding to 14% of the measured magnetic field amplitudes.

## Summary and Outlook

In this study, we highlight several results from joint observations by Van Allen Probes and Arase. Many additional studies using data from these satellites are being conducted and will be published in the near future. The Van Allen Probes mission was successfully completed in October 2019. During the period of joint observations by the three spacecraft (2017–2019), 23 magnetic storms, including major storms in July and September 2017 and August 2018, were observed. The configuration of the satellite locations is different for each event and thus, various new results are expected from these observations.

Joint observations by Van Allen Probes and Arase contributed significantly to addressing key questions about the inner magnetosphere, which have never been achieved by a single satellite observation. Multi-point measurements identified the spatial distributions of wave–particle interactions that cause electron transport and acceleration as well as the loss processes and MLT distributions of low-energy ions. Multi-point measurements at different latitudes at almost the same field line are important for studying wave propagation along the field line. Sequential observations with multi-spacecraft can discriminate between spatial and temporal variations among electron flux variations. In addition to multi-point observations by Van Allen Probes and Arase, intercalibration using the observational data from both missions allows us to combine these datasets with uniform qualities.

**Fig. 18:**
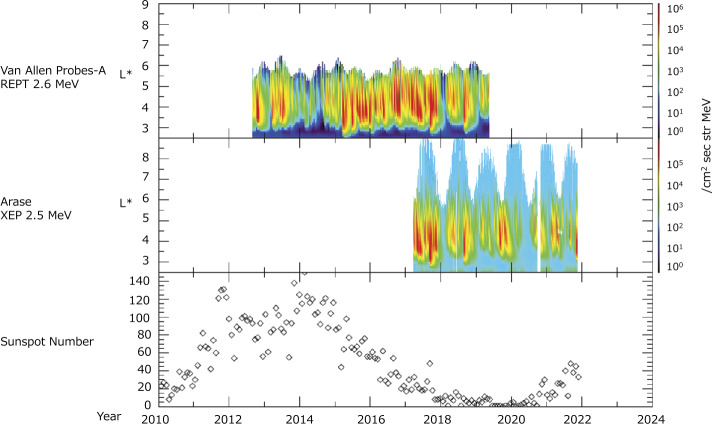
$L$-time diagram of relativistic electrons measured by Van Allen Probes/REPT (2.6 MeV) and Arase/XEP (2.5 MeV). The bottom panel indicates the sunspot-number

The Van Allen Probes mission radically changed our understanding of the radiation belts and inner magnetosphere, local particle energization, particle loss, ring current generation, and other important phenomena. The Van Allen Probes cover the rising phase of solar cycle 24 and the declining phase. Arase provided observations from March 2017, corresponding to the late declining phase of the cycle. Figure 18 shows the $L$-time diagram of 2.5 MeV electrons measured by the Van Allen Probes/REPT and Arase/XEP. Semiannual variations (Baker et al. 1999; Miyoshi et al. 2004) and solar cycle variations (Miyoshi et al. 2004; Miyoshi and Kataoka 2011; Li et al. 2006) of relativistic electrons were clearly observed. During severe magnetic storms, large flux enhancements were observed in the low $L$-shell region.

After March 2017, similar variations were detected by the Van Allen Probes and Arase. Because the inclination angle of Arase is larger than that of the Van Allen Probes, Arase observed a higher $L$-shell ($L \sim 10$). After October 2019, Arase has continued operations to provide comprehensive observations of plasma, particles, fields, and waves in the inner magnetosphere; further, variations in the outer belt electrons were observed after the Van Allen Probes era. As discussed, the intercalibration between the Van Allen Probes and Arase is essential for the seamless analysis of both satellite data. Therefore, overlapped periods are a unique opportunity to obtain uniform-quality datasets from both satellite data. By combining the data from Van Allen Probes and Arase, a full-cycle 11 year observation of the outer belt (2012–2023) will be realized soon, which will be the first comprehensive observation of the inner magnetosphere and radiation belts.

## Data Availability

LEPe L2 omni flux v02-02 (Wang et al. 2018: 10.34515/DATA.ERG-04002) LEPi L2 omni flux v03 (Asamura et al. 2018b: 10.34515/DATA.ERG-05001) MEPe L2 omni flux v01-02 (Kasahara, Yokota et al. 2018b: 10.34515/DATA.ERG-02001) MEPi L2 omni flux v02-00 (Yokota et al. 2018: 10.34515/DATA.ERG-03001) HEP L2 omni flux v03-01 (Mitani et al. 2018: 10.34515/DATA.ERG-01001) XEP L2 omni flux v01-00 (Higashio et al. 2018: 10.34515/DATA.ERG-0001) PWE-HFA L3 v01-01 (Kasahara, Kumamoto et al. 2018: 10.34515/DATA.ERG-10001) Arase OBT L2 v03 (Miyoshi, Shinohara and Jun 2018: 10.34515/DATA.ERG-12000) Sym-H index (World Data Center for Geomagnetism, 2022: 10.14989/267216)
